# *In silico* assessment data of allergenicity and cross-reactivity of NP24 epitopes from *Solanum lycopersicum* (Tomato) fruit

**DOI:** 10.1016/j.dib.2018.09.074

**Published:** 2018-10-03

**Authors:** Majeed Jamakhani, S.S. Lele, Bhagwan Rekadwad

**Affiliations:** a Department of Food Engineering and Technology, Institute of Chemical Technology, Nathalal Parikh Marg, Matunga (E), Mumbai 400019, India; b National Centre for Microbial Resource, National Centre for Cell Science, NCCS Complex, Savitribai Phule Pune University Campus, Ganeshkhind Road, Pune 411007, Maharashtra, India

**Keywords:** NP24 protein, Epitope mapping, Fruit and vegetable allergies, Oral allergy syndrome, IgE antibody

## Abstract

This paper describes data on allergies caused by food (vegetable) and their negative impact on the nutritional balance of the human body. Allergic responses to vegetables such as tomatoes, capsicum and spinach are next to fish, eggs and nuts. Epitopes such as NP24 (allergens) are one of the salt-induced allergenic proteins found in the thaumatin-like protein (TLP) family. The mechanism of allergenicity of TLP found in *Solanum lycopersicum* (Tomato) fruit is poorly studied. Here we demonstrated allergenicity conferred by the NP24 protein found in Tomato. The data on the cross-reactivity of NP24 protein was generated using Allergen Online and Allermatch tools. Tomato allergenic protein epitope shows a significant identity of with allergens reported in Capsicum, Olive, Kiwi, Tobacco and Banana allergens. Hence, the datasets of sequences, comparative analysis and homology epitope mapping over three dimensional (3D) structures revealed that NP24 has higher cross-reactivity to Capsicum and Tobacco proteins. Thus, this data probably act as limelight for planning wet lab experiments.

**Specifications table**

**Table:** 

Subject area	*Biotechnology*
More specific subject area	*Proteomics, Bioinformatics*
Type of data	*Tables, images figures*
How data was acquired	*In silico analysis*
Data format	*Both, raw and analyzed*
Experimental factors	*As specified and recommended by tools and algorithm*
Experimental features	*Structural and functional analysis of protein NP24*
Data source location	Department of Food Engineering and Technology, Institute of Chemical Technology, Matunga (E), Mumbai, India
Data accessibility	*Primary data is available in NCBI repository portal. Analysed data is available within this article.*
Related research article	Y. Kondo, A. Urisu, R. Tokuda, Identification and Characterization of the Allergens in the Tomato fruit by Immunoblotting, Int. Arch. Allergy Immunol. 126 (2001) 294–299.
	R. Pressey, Two isoforms of NP24: a thaumatin-like protein in Tomato fruit, Phytochemistry. 44, 1241–1245.

**Value of the data**

• This data depicts a method for the detection of severe allergic reactions caused by an allergic protein found in plants. This result in the release of histamine. It would be an indicator of IgE antibodies bound by tomato allergens.
• Data gives in-depth information on allergic NP24 protein found tomato which shows top identity with bell pepper, kiwi and olive TLPs.
• This method will be useful for prediction of IgE epitopes in food, vegetables and pollen TLPs laid in epitopes (either region 1 and 2). This work would act as a limelight for planning wet lab experiments.

## 1 Data

This paper describes the data on plant proteins (PR1 to PR 17) especially belonging to vegetables. Allergic plant protein from *Solanum lycopersicum* (Tomato) such as Thaumatin protein belonged to PR-5 group. It shows functional diversity in allergenicity and kinase function. Tomato fruit NP24 protein has seven different types of IgE epitopes. Mapping of IgE epitopes of NP 24 protein shows that NP 24 protein is allergic and shows cross-reactivity as well.

Nutritious food is required for growth, development and maintenance of health. Most commonly used food such as fishes, eggs, fruits and nuts have wide acceptability and fall under most nutritious foods. On contrary, intake of such food can cause allergy or create allergic situations in some individuals. Overreaction of body immune defence system is the cause for allergies. A study of the prevalence of sensitization to foods in Europe was carried out with 4522 individuals living in 13 countries for IgE test against 24 foods. The survey reported that individuals from most of the countries the high prevalence of vegetables, fruits and nuts than to eggs, milk and kinds of seafood. Allergic sensitivity to nuts was 7% whereas 0.2 and 0.4% to fish and eggs respectively. Similarly, Tomato is one of the most commonly consumed vegetables across the next to apple and wheat. “*Solanum lycopersicum* fruits that are called as vegetables by “Nutritionists”. Food allergy from Tomato fruit showed the significant allergic prevalence of 3.3% [1]. Prevalence of clinical oral food challenge (OFC proven) for food allergy test in preschool children in developed countries was reported to be as high as 10%. Unlike in developed countries, it is 7% in Asian countries such as China and India [2]. However, urbanization increased consumption of processed food and stressful lifestyle has resulted in reduced immunity and increased allergies to foods especially in children [3], [4].

The hypersensitive response is one of the most efficient mechanisms for conferring immunity a from phytopathogens which include fungi, bacteria and viruses. The pathogen-related proteins (PR proteins) are defensive molecules which protect plants. These PR proteins are belonging to the family of "stress-inducible" proteins. These were first discovered in *Nicotiana tabacum* (Tobacco) plants causing hyper sensitively to infection from Tobacco Mosaic Virus [5], [6]. Later, many PR proteins have been detected in other plant species [7], [8], [9], [10], [11].

The pathogenesis-related protein families are broadly classified into 17 groups. Amongst them, thaumatin-like proteins (PR-5) is the fifth group of the PR protein family having molecular weights ranged from 20 to 26 kDa. They were named as thaumatin-like proteins because the amino acid sequence is homologous to thaumatin-a sweet-tasting protein derived from *Thaumatococcus daniellii* [12]. Thaumatin and Thaumatin-like proteins also identified in animals [13] and fungi [14], [15]. The thaumatin family protein has eight disulphide residues [16], [17]. Despite the lack of atopic individuals (in humans), pollens and food TLPs are identified as inhalants and ingestant respectively [18]. Reports show that 39.2% of children are monosensitized to grass pollen and has allergy to Tomato fruits IgE antibodies [19]. In 1988 Ortolani et al., confirmed that association between Tomato fruit oral allergy syndrome (OAS) and grass pollen allergy is statistically significant [20]. It has also been reported that sometimes anaphylaxis arises within few hours as soon as Tomato fruit consumed [21]. Several allergens in Tomato fruit have been described such as Sol-l1, Sol-l2, Sol-l3, Sol-l chitinase, Sol-l-Glucanase, Sol-l-peroxidase and Sola-TLP (NP24). But, the clinical relevance of each of these allergens is yet not clear. Only a limited number of TLPs have been identified from plant pollens and foods. Among fruits, only hybrid forms of TLPs are shown a hypertensive reaction with IgE [22], [23], [24]. So far, out of 15 allergen TLPs, only 7 TLPs have been crystallized and their 3D structures were elucidated [25]. However, comparative analysis of the structural features of allergenic TLPs in the context of prediction of IgE epitopes have not been reported so far.

The salt-induced TLPs is Protein NP24 (molecular weight 24 kDa) containing 247 amino acids is found in Tomato fruit tissues [26]. Previously NP24 was first isolated, purified and crystallized from Tomato fruit fruits [27]. Later studies reported that there are two isoforms (I and II) of the thaumatin-like protein NP24 present [19]. Isoform-I was expressed mainly in the outer pericarp of healthy Tomato fruit fruits and low in green Tomato fruits which subsequently increases during ripening of fruit. On the other hand, Isoform-II is relatively high in green Tomato fruits. It׳s concentration rise as the fruit turns pink and subsequently decreases as the fruit turns red. Fully ripened Tomato fruit (mature fruit) will have mainly isoform-I and the half-ripened fruit will have both isoforms (I & II) in significant quantity.

Detection allergy in individuals either in vivo or in vitro using molecular biology techniques is very difficult task time-consuming task and cost ineffective as well [28]. Possible development of severe allergic risks during the test and lack of sensitivity of allergic reaction are the major drawbacks of in vivo and in vitro methods. These shortcomings make the computational method as a good approach for the identification of epitopes and allergenicity. From the above discussion, it may be inferred that vegetable allergies, especially by consumption of Tomato fruit are much more prevalent than what one would expect. Hence the present work was undertaken mainly for computational analysis of NP24 protein from Tomato fruits in order to identify allergic components such as IgE epitopes, their position and possible cross-reactivity with other food and pollen TLPs.

## 2 Experimental design, materials, and methods

### 2.1 The protein sequence data of the NP24/Thaumatin-like protein

The NP24 protein sequences were retrieved from the National Center for Biotechnology Information (https://www.ncbi.nlm.nih.gov/). Search result yielded around 5, 359 hits from different organisms. Further narrowing down to *Solanum lycopersicum* yielded 44 results. We have used the complete gene sequence which has 247 amino acids (NCBI accession number P12670).

### 2.2 Prediction of cross reactivity of NP24 protein

The cross-reactivity of Protein NP 24 sequence determined using freewares such as Allergen online (www.allergome.org) and Allermatch (http://allermatch.org/) tools. Both online tools give comparative data on cross-reactivity and IgE binding properties of clinically important NP24 protein.

### 2.3 Prediction of 3-dimensional (3D) confirmation of NP24 protein

The HHpred method was used to predict the 3D structure of homologous sequences. HHpred utilizes Hidden Markov Model – Hidden Markov Model (HMM-HMM) algorithm/ (http://toolkit.tuebingen.mpg.de/) for identification of 3D structure. This modeling process involves various steps such as (a) Database search and E value: Here homologous sequences detected greater than 90% and very less E value are considered, (b) To check similarity between secondary structure of sequences, (c) Identification of possible conservative motifs for designing structure, (d) Aligning the target sequence with the template structure, and (e) Realignment of sequences.

### 2.4 Data on prediction and characterization of antigenic determinants of NP24 protein

Antigenic determinants are the part of an antigen. These are identified by the antibody, B cells or T cells, react with them and produce hypersensitivity reactions. Prediction of linear B cell with accuracy is a challenging process to design immunotherapy. BepiPred prediction method adopted for identification and locating B cell epitopes by a combination of Hidden-Markov model and Parker & Levitt propensity scale algorithm. Algpred tool (http://www.imtech.res.in/raghava/algpred/submission.html) used for the prediction of antigenic determinant binding to IgE. To predict allergens, initially, BLAST was used to identify sequences and aligned using Allergen Representative Peptides. Multiple Em for Motif Elicitation (MEME) tool was used for discovering motifs in a group of related protein sequences followed by statistical analysis. Phylogenetic tree analysis was done using *MEGA6*.

### 2.5 IEDB homology mapping of NP24 protein

Mapping gives the best representation of antigenic determinant points over the 3D structure. It also provides the information about the position of epitopes i.e. whether epitope regions lies inside the structure or at the surface level and how this epitope distributed over the similar sequences as that of the query sequence. IEDB epitope homology mapping tool (http://tools.immuneepitope.org/tools/bcell/iedb) detects PDB that are homologous to the epitope source sequence.

### 2.6 Epitope Conservancy Analysis (ECA)

ECA tool computes the degree of conservancy of an epitope within a given protein sequence set at a given identity level. Obtained results were represented as summary view (for all epitope sequences) and a detail view (for individual epitope). The summary view for each epitope shows degree of conservancy (percentage of protein sequence matches a specified identity level) and the matching minimum/maximum identity levels within the protein sequence set. The detail view of an epitope shows the positions and the matching protein sub-sequences for all sequences in the protein dataset.

## 3 Datasets of cross-reactivity of NP24 protein

Allergen Online, Allermatch and FARRP tools database gave significant information regarding cross-reactivity. In 2000 Alberse hypothesized that greater than 70% identity by comparing a query sequence with homologous known allergen sequences showed the significant cross-reactivity. While those with less than 50% identities are unlikely to be cross-reactive. This suggests that alignment of the query with greater than 50% identity with allergen sequence were cross-reactive in full-length alignment. In 80-mer sliding window method, similarity search was performed for every 80-amino acids segment of the query sequence. The cutoff value was greater than 35% (as per FAO/WHO 2001 expert panel recommendation) which indicates the possible cross-reactivity of allergens. However, in the 8-amino acid exact match (8-mer) method, any exact match for the query was considered to identify the protein as a potential cross-reactive allergen. Cross-reactivity analysis using all three methods in both FARRP & Allermatch gives in-depth cross-reactivity information.

Table 1 shows the results of allergen online database. In full-length alignment method for allergic food, Kiwi, Olive and Sapota showed significant identity to NP24 (above 65%), whereas allergic pollen like Japanese Cedar and White Cedar showed an identity of approximately 50%. In-80-slide window result for protein NP24, the number of 80-mer sequences was found to be 168 among that 29 sequences showed matching of 80 amino acid stretches with the allergens deposited in databases. In the 8-mer, the total number of 8-mers were 240. Of which, 15 sequences with at least one 8-mer match corresponding to allergens found in the database.

**Table 1:** Full-length (FL) alignment method lists allergen matches with > 50% identity, 80-mer match method lists allergen matches with > 35% identity, and 8-mer match method lists allergens with at least one exact match.

**Hit**	**Description**	**Species**	**Full length**	**Sliding 80-mer**	**8-mer**
			Hits %	Hits %	Hits %
1	gi|146737976|gb|ABQ42566.1| thaumatin-like protein	*Actinidia deliciosa*	68.8	65.9	9
2	gi|269996497|gb|ACZ57583.1| allergenic thaumatin [	*Olea europaea*	64.1	68.8	12
3	gi|349503011|gb|AEP84104.1| acidic thaumatin-like	*Manilkara zapota*	65	64.1	19
4	gi|139002766|dbj|BAF51970.1| thaumatin-like protei	*Cryptomeria japonica*	52.8	65	5
5	gi|9087177|sp|P81295.1|PRR3_JUNAS RecName: Full=Pa	*Juniperus ashei*	54.1	52.8	3
6	gi|38456224|gb|AAR21072.1| PR5 allergen Jun r 3.2	*Juniperus rigida*	53.6	54.1	2
7	gi|51316532|sp|Q9LD79.2|PRR3_JUNVI RecName: Full=P	*Juniperus virginiana*	58.2	53.6	0
8	gi|38456222|gb|AAR21071.1| PR5 allergen Jun r 3.1	*Juniperus rigida*	53.1	58.2	2
9	gi|38456228|gb|AAR21074.1| PR5 allergen Cup s 3.2	*Cupressus sempervirens*	52.6	53.1	2
10	gi|38456230|gb|AAR21075.1| PR5 allergen Cup s 3.3	*Cupressus sempervirens*	52.6	52.6	2
11	gi|135917|sp|P27357.1|TLP_WHEAT RecName: Full=Thau	*Triticum aestivum*	45.9	52.6	1
12	gi|9929163|emb|CAC05258.1| Cup a 3 protein [Hesper	*Cupressus arizonica*	52.2	45.9	2
13	gi|190613911|gb|ACE80959.1| putative allergen Pru	*Prunus dulcis, Prunus persica*	42	52.2	0
14	gi|25091405|sp|P83332.1|TLP1_PRUPE RecName: Full=T	*Prunus persica*	41.6	42	0
15	gi|190613909|gb|ACE80958.1| putative allergen Pru	*Prunus dulcis, Prunus persica*	41.6	41.6	0
16	gi|190613907|gb|ACE80957.1| putative allergen Pru	*Prunus dulcis, Prunus persica*	41.6	41.6	0
17	gi|60418848|gb|AAX19851.1| thaumatin-like protein	*Malus, domestica*	42	41.6	0
18	gi|218059715|emb|CAT99611.1| thaumatin-like protei	*Malus, domestica*	42.9	42	0
19	gi|60418842|gb|AAX19848.1| thaumatin-like protein	*Malus, domestica*	42	42.9	0
20	gi|392507603|gb|AFM77001.1| pathogenesis related p	*Malus, domestica*	46.2	42	0
21	gi|30316292|sp|Q9FSG7.1|TP1A_MALDO RecName: Full=T	*Malus, domestica*	42	46.2	0
22	gi|218059718|emb|CAT99612.1| thaumatin-like protei	*Malus, domestica*	42.9	42	0
23	gi|190613905|gb|ACE80956.1| putative allergen Pru	*Prunusdulcis, Prunus persica*	41.6	42.9	0
24	gi|359744030|gb|AEV57471.1| thaumatin-like protein	*Prunus persica*	40.7	41.6	0
25	gi|1144346|gb|AAB38064.1| thaumatin-like protein p	*Prunus avium*	41.4	40.7	0
26	gi|25091406|sp|P83335.1|TLP2_PRUPE RecName: Full=T	*Prunus persica*	42	41.4	1
27	gi|190613903|gb|ACE80955.1| putative allergen Pru	*Prunus dulcis, Prunus persica*	42	42	1
28	gi|190613941|gb|ACE80974.1| putative allergen Pru	*Prunus dulcis*	41.6	42	1

In full-length alignment method, interestingly food alignment window for protein NP24 shows more than 50% identity to TLPs Capsicum, Kiwi fruit, Banana, Apple, White Cedar and *Cupressus sempervirens*. In 80-slide window and exact match method result for protein NP 24 showed the similar result as of full length (Table 2).

**Table 2:** Allergic assessment of NP24 protein using Allermatch Tool: a] Results of FASTA alignment of input sequence against UniProt and WHO-IUIS database. (Number indicates to percentage identity). b] (i) Percent identical amino acids in the aligned 80-aa sliding window, (ii) the number of hits the input sequence had with this allergen, and (iii) the percentage of windows analyzed for this input sequence hitting this allergen iv) Results of a FASTA alignment of the complete input sequence against this database sequence. The first number is the percentage of identity. The second number is the length of sequence over which FASTA aligned c] the percentage of exact hits the input sequence is found to hit this allergen sequence.

**Protein ID**	**Species Name**	**Full length**^**a**^**(%)**	**80 merwindow**^**b**^**(%)**				**6 amino acid match**^**c**^**(%)**
			**I**	II	III	IV	
Q9ARG0	*Capsicum annuum* (bell peper)	88.98	95	168	100	88.99	49.17
P81370	*Actinidiadeliciosa* (kiwi)	69.26	73.75	168	100	69.27	8.68
O22322	*Musa acuminata* (banana)	62.96	73.75	144	85.71	62.96	6.2
P81295	*Juniperusashei* (white cedar)	54.16	63.75	168	100	54.15	3.72
Q9LD79	*Juniperusvirginiana* (red cedar)	59.77	62.5	67	39.88	59.77	1.65
Q69CS2	*Cupressussempervirens* (cypress)	52.63	61.25	168	100	52.63	2.48
Q69CS3	*Cupressussempervirens* (cypress)	52.63	61.25	168	100	52.63	2.48
B6CQT7	*Prunuspersica* (peach)	42.66	50	151	89.88	42.67	0.83
B6CQT5	*Prunuspersica* (peach)	42.22	48.78	152	90.48	42.22	0.83
Q9FSG7	*Malusdomestica* (Apple)	42.66	48.75	155	92.26	42.67	0.83
P50694	*Prunusavium* (cherry)	41.77	46.67	136	80.95	41.78	1.65
B6CQT3	*Prunuspersica* (peach)	42.34	46.34	147	87.5	42.34	2.89

**Table 3:** IgE epitopes of Homologous with NP 24 protein and their matched respective sequences.

**Species Name**	**PDB structures**	**IgE epitopes**			**PI**	**MW**
		**IgE epitope sequence**	**Matched sequence**	**Position**		
*Capsicum annuum* (Q9ARG0)	NOT available	AAGTASARFWGRT	PPGTAMARIWGRT	58	7.53	23,984.78
		TFDASGKGSCQTG	NFDGSGRGSCQTG	73		
*Actinidia deliciosa* ( Kiwi) (P81370)	4bct	AAGTASARFWGRT	GAGTKGARVWPRT	60	7.91	21,614.23
		ADINAVCPSELK	ADINGQCPNELR	148		
		TFDASGKGSCQTG	NFDGAGRGKCQTG	75		
		VDGGCNSACNVFKT	APGGCNNPCTVFKT	160		
*Musa acuminate* (Banana) (O22322)	1z3q	AAGTASARFWGRT	NAGTTGGRIWGRT	62	6.68	20,429.05
		TFDASGKGSCQTG	SFDGSGRGRCQTG	77		
*Juniperusashei* (Mountain cedar) (P81295)	1kur	AAGTASARFWGRT	AAGTASARFWGRT	62	4.79	20,994.34
		ADINAVCPSELK	ADINAVCPSELK	146		
		TFDASGKGSCQTG	TFDASGKGSCQTG	77		
		VDGGCNSACNVFKT	VDGGCNSACNVFKT	158		
*Prunus persica* (Peach) (B6CQT3)	NOT available	ADINAVCPSELK	ANVNLVCPSELQ	154	4.81	25650.06
*Cupressussem pervirens* (Q69CS2)	NOT available	AAGTASARFWGRT	AAGTASARFWGRT	62	5.32	21103.51
		ADINAVCPSELK	ADINAVCPSELK	146		
		TFDASGKGSCQTG	TFDASGKGSCRSG	77		
		VDGGCNSACNVFKT	VDGGCNSACNVLQT	158		
*Cupressussem pervirens* (Q69CS3)	NOT available	AAGTASARFWGRT	AAGTASARFWGRT	62	5.32	21,103.51
		ADINAVCPSELK	ADINAVCPSELK	146		
		TFDASGKGSCQTG	TFDASGKGSCRSG	77		
		VDGGCNSACNVFKT	VDGGCNSACNVLQT	158		
*Juniperus virginiana* (Q9LD79)	NOT available	Not available	NOT available	NOT available	7.77	9517.51
*Prunus avium* (P50694)	2AHN	ADINAVCPSELK	ANVNAVCPSELQ	157	4.84	25,706.97
*Prunus persica* (B6CQT5)	NOT available	ADINAVCPSELK	ADINKVCPAELQ	158	5.13	25,840.16
*Prunus persica* (B6CQT7)	NOT available	ADINAVCPSELK	ADINKVCPAPLQ	158	8.31	23,337.33
*Malus domestica* (Q9FSG7)	3zs3	AAGTASARFWGRT	APSPWSGRFWGRT	67	4.72	23,210.91
*Alternaria alternate* (P79085)	3vor, 4aud	KISEFYGRKP	KISEFYGRKP	41	4.75	16,979.93
		YSCGENSFMD	YSCGENSFMD	87		
		YYNSLGFNIK	YYNSLGFNIK	54		
*Solanum lycopersicum* (P12670)	Query sequence	AAGTASARFWGRT	PRGTKMARIWGRT	58	8.28	26,646.21
		TFDASGKGSCQTG	NFNAAGRGTCQTG	73		

## 4 Recognition and assessing antigenic determinants of NP24 protein

**Fig. 1:**
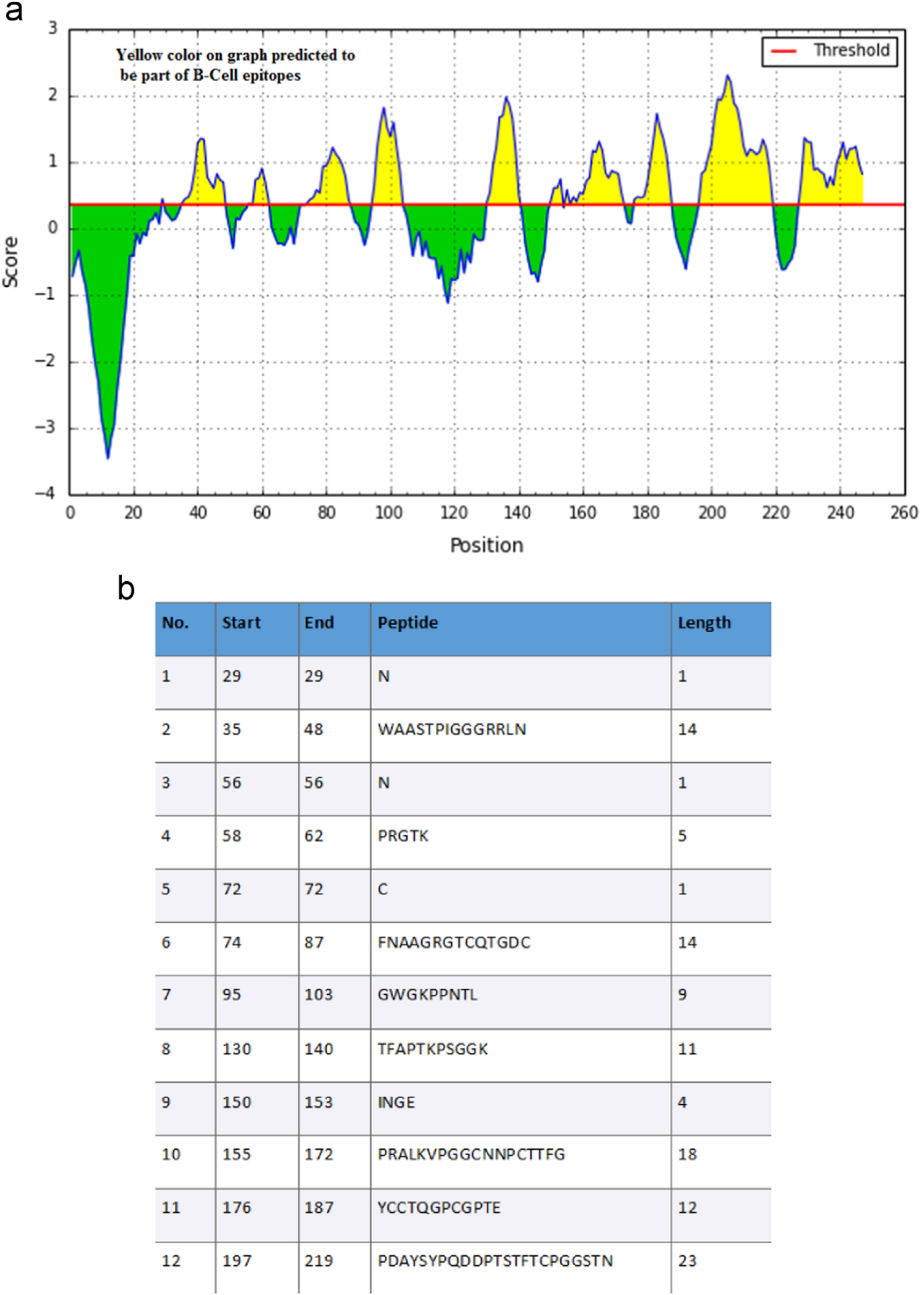
(a) Protein NP24 B-cell epitopes predicted showed in yellow peaks. (b) Starting and ending positions of 12 predicted epitopes showed: Analysis of NP24 protein by Algpred tool revealed the presence of two IgE epitopes viz., ‘IgE epitope 1’, stretching from position 58–70 (PRGTKMARIWGRT) and ‘IgE epitope 2’, stretching from position 73–85(NFNAAGRGTCQTG) as shown in Fig. 2. Both IgE Epitope surfaces overlap with the thaumatin family signatures. Further, B-cell epitope residues with the high score (FNAAGRGTCQTGDC) and medium scores (PRGTK) were found in IgE epitopes 1 and 2, which indicate their higher accessibility for antibody recognition.

NP24 protein sequence was retrieved from the SwissProt database (accession number P12670) and analyzed for B cell epitopes using IEDB tool. Prediction scores for each residue of NP24 obtained by IEDB BepiPred (Fig. 1). The residues with scores above the cutoff value (>35%) was predicted as epitope and highlighted in yellow color. Total 12 epitopes were identified. Amongst 12 epitopes, epitope number 10, 6, 2 and 11 have shown high scores followed epitope 8, 7 and 4 (with intermediate scores) which followed by epitope 1, 3, 5, 9 and 12.

## 5 Epitope conservancy analysis and distribution of epitopes

Epitope conservancy analysis tool calculates the degree of conservancy of an epitope within a given protein sequence set at a different degree of sequence identity. The degree of conservation is defined as “the fraction of protein sequences containing the epitope at a given identity level”. Epitome conservancy analysis was performed on 7 identified epitopes from Algpred tool. It was observed that IgE Epitopes 1, 3 & 2 showed the highest degree of the conservancy. Detailed analysis is depicted in Table 4 shows epitope sequence, starting position, ending position and percentage of identity of the query sequence. Epitope regions of NP24 predicted by different tools as discussed earlier. Table 5 shows positions 50 to 73 of the NP24 sequence.

**Table 4:** Distribution of Epitopes and their conservancy among sequences.

**Epitope No.**	**Epitope name**	**Sequence of epitope**	**Length of epitope sequence**	**Percentage of protein sequence matches at identity ≥ 100%**	**Minimum identity**	**Maximum identity**
1	1	AAGTASARFWGRT	13	28.57% (4/14)	38.46%	100.00%
2	2	TFDASGKGSCQTG	13	14.29% (2/14)	30.77%	100.00%
3	3	ADINAVCPSELK	12	21.43% (3/14)	25.00%	100.00%
4	4	VDGGCNSACNVFKT	14	7.14% (1/14)	28.57%	100.00%
5	5	KISEFYGRKP	10	7.14% (1/14)	30.00%	100.00%
6	6	YSCGENSFMD	10	7.14% (1/14)	20.00%	100.00%
7	7	YYNSLGFNIK	10	7.14% (1/14)	30.00%	100.00%

**Table 5:** Epitope regions of NP24 predicted by using bioinformatics tools.

**Tools**	**Epitope Types**	**Epitope Regions obtained**			
DNA Star	Bcell	6–10, 17–40,46–80, 103–106, 138–166, 173–207			
	T cell-AMPHI	22–28, 38–48, 64–85, 87–94, 98–105, 132–148, 164–176,178–179			
	Antigenicity- jameson-wolf	5–9, 21–30, 36–41, 56–67, 78–81, 112–119, 129–138, 140–142, 171–177, 181–187			
	MHC-II epitopes	16–21			
ABC prediction	B cell	38–53, 13–28, 131–146, 175–190, 4–19, 125–140, 119–134, 73–88,58–73, 1–8, 194–207,155–170,67–82,47–62			
IEDB	Bipred	15–28, 38–42, 54–67, 75–83, 110–120, 130–133, 135–152, 156–167, 177–199			
	Algpred	**IgE epitope**	**Sequence-matched**	**Position**	**PID**
		AAGTASARFWGRT	PRGTKMARIWGRT	58	61.53846
		TFDASGKGSCQTG	NFNAAGRGTCQTG	73	61.53846

## 6 Phylogenetic analysis of NP24 protein

**Fig. 2:**
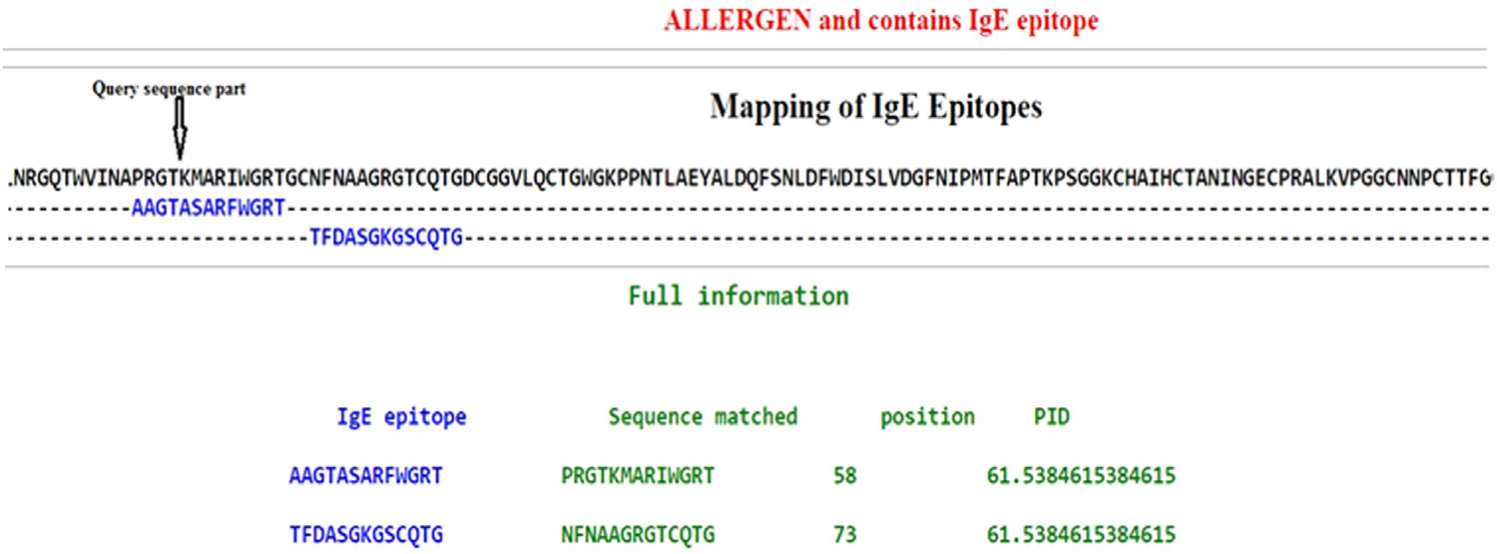
Phylogenetic analysis of NP24 protein using *MEGA6 software*. The evolutionary history was inferred using the Neighbor Joining method. The evolutionary distances were computed using the Jukes-Cantor method. The percentage of replicate trees in which the associated taxa clustered together in the bootstrap test (1000 replicates).

**Fig. 3:**
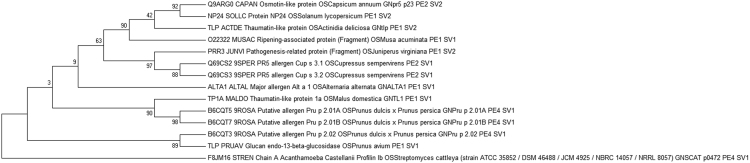
IgE epitope of Protein NP 24 by Algpred: Starting position in sequence for epitope1 and Epitope 2 showed as 58 & 73 respectively. Algpred tool was used to analyze the allergenicity of our protein NP24 and its homologous proteins, results are shown in Table 3. Analysis of proteins NP24 suggest that there are 7 different types of IgE epitopes found in the Homologous sequences namely Epitope 1 (AAGTASARFWGRT), Epitope 2 (TFDASGKGSCQTG), Epitope 3 (ADINAVCPSELK), Epitope 4 (VDGGCNSACNVFKT), Epitope 5(KISEFYGRKP), Epitope 6 (YSCGENSFMD) and Epitope 7 (YYNSLGFNIK). Few interesting findings of these predictions are as follows; 1) IgE epitopes ranges from 0 to 4 that is some protein sequences have 0 epitopes and some have maximum 4 epitopes. 2) IgE epitopes present in Protein NP 24 showed 42.85% presence in other homologous sequences, i.e. epitope 1 and epitope 2. 3) 13/14 (92.85%) sequences displayed either one or 4 IgE epitopes. 4) 1/14 (7.2%) displayed zero IgE epitope.

The phylogenetic tree was inferred using MEGA6 tool to understand the evolutionary pattern of homologous sequences which share IgE epitopes with NP24 (Fig. 2). Homologous sequences obtained from epitope conservancy were alignment by using MUSCEL. The phylogenetic tree was constructed by *MEGA 6.0* tool. Protein NP24 is closely related to osmotic like protein of *Capsicum annum* (Fig. 3) sharing the Epitope 1 & 2. This infers that the person with Tomato fruit allergy might have an allergy to Capsicum.

## 7 Data on computational modeling of NP24 protein

**Fig. 4:**
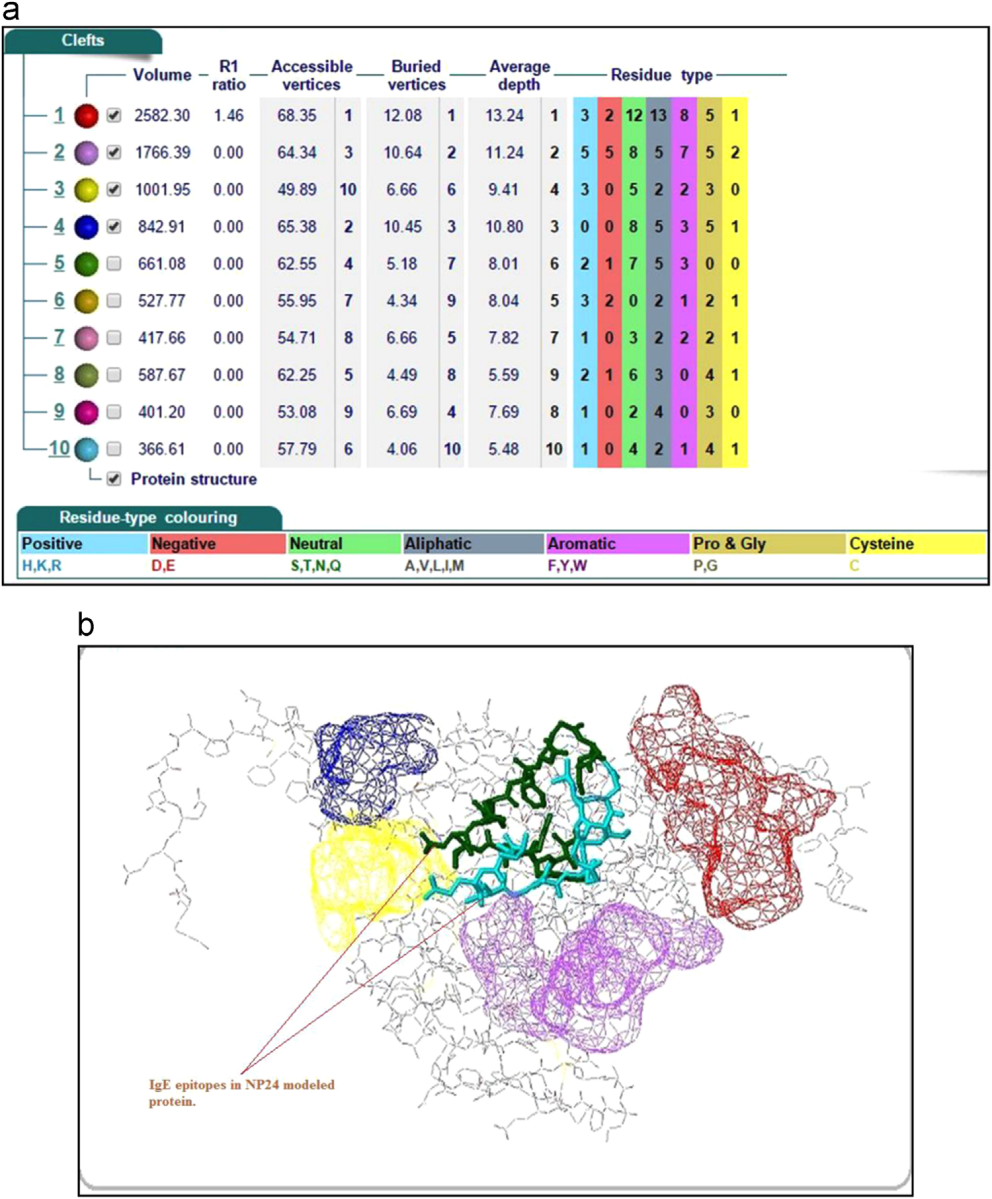
(a) Cleft numbering 1–10 was assigned based on its volume, highest being 1 indicated in red color, next to purple. (b) Protein NP24 showing IgE epitopes was predicted by AlgPred; the structure was shown as a cartoon. IgE epitopes 1 and 2 were shown in cyan and green stick shapes.

Crystal structure of NP24-I (PDB ID: 2i0w) was created using X-ray diffraction, at a resolution of 2.5 Å and deposited in PDB in 2006. An in silico modeling of NP24-I was compared with the crystal structure of NP 24 using DALI tool. Homology method was used to develop the model of Protein NP24. Total 11 crystallographic structures of TLPs were available in RCSB PDB database. In order to construct the structure of NP24, one can choose any one of these or a combination of these structures as templates. On the basis of alignment score and coverage of sequences, 6 structures were selected namely- cherry allergen Pru av 2(2AHN), Pathogenesis-related protein 5d from *Nicotiana tabacum* (1AUN), kiwi-fruit allergen Act d 2 (4BCT), Zeamatin (1DU5), Thaumatin I (2VHK) & thaumatin-like xylanase inhibitor TLXI (3G7M). The further 3D model was generated using Modeller of HHpred (Fig. 4).

**Fig. 5:**
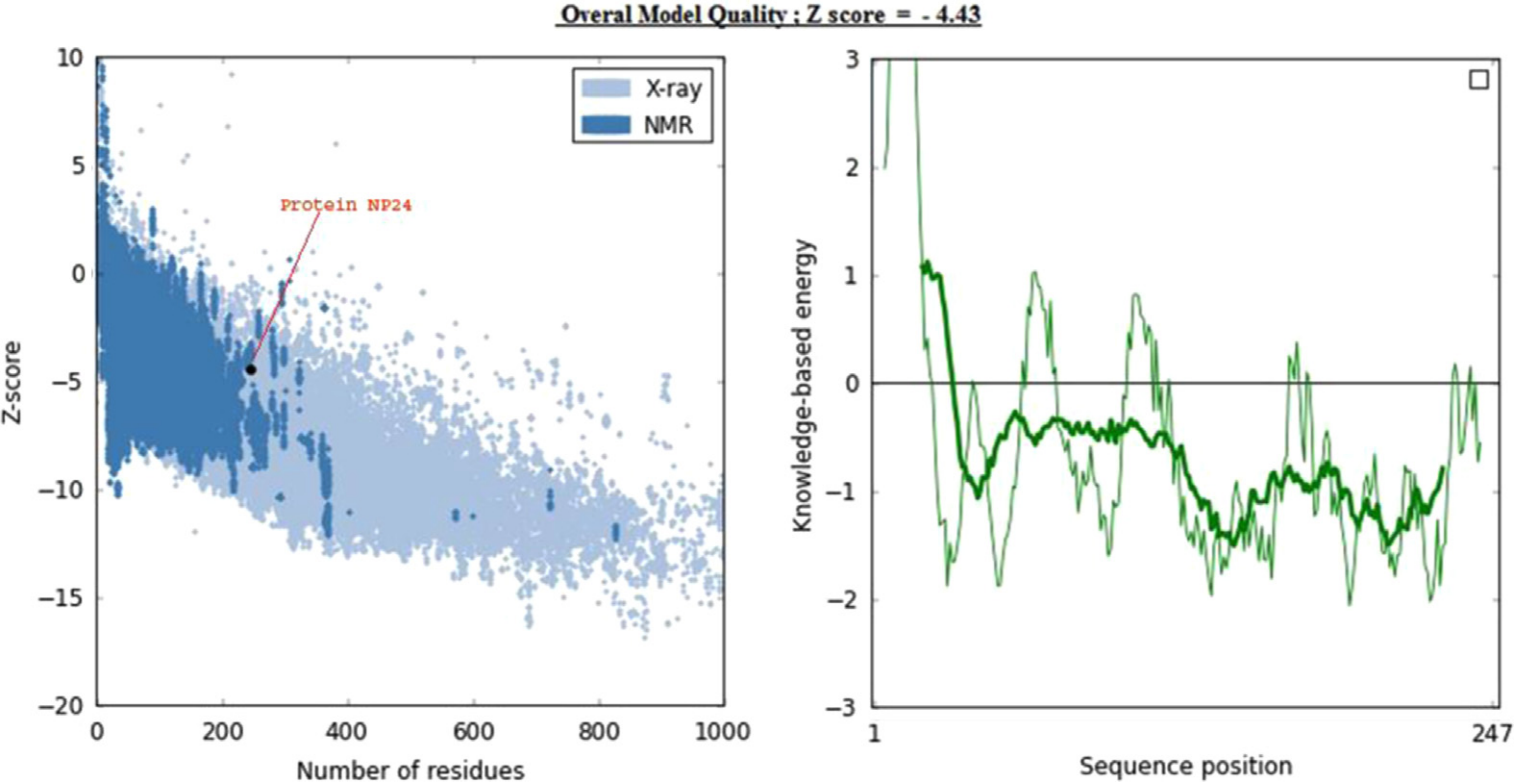
NP24 protein Model Quality: Left Plot shows overall model quality which lies within the range of scores typically found for native proteins of similar size while right plot shows local model quality by plotting energies as a function of amino acid sequence position. In general, positive values correspond to problematic parts of the input structure.

**Fig. 6:**
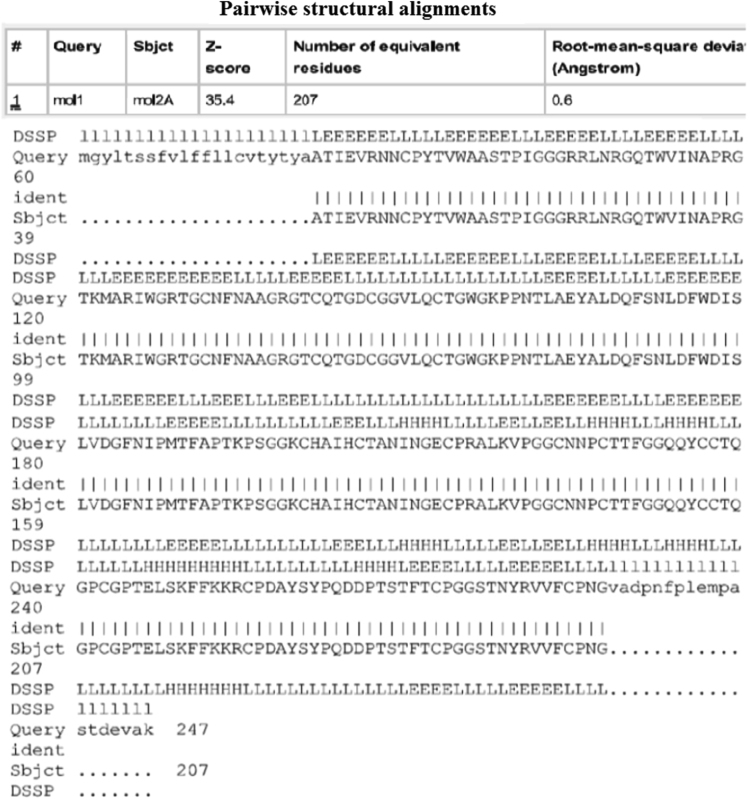
Dali structural alignment between crystallographic structure and predicted structure of NP24.

**Fig. 7:**
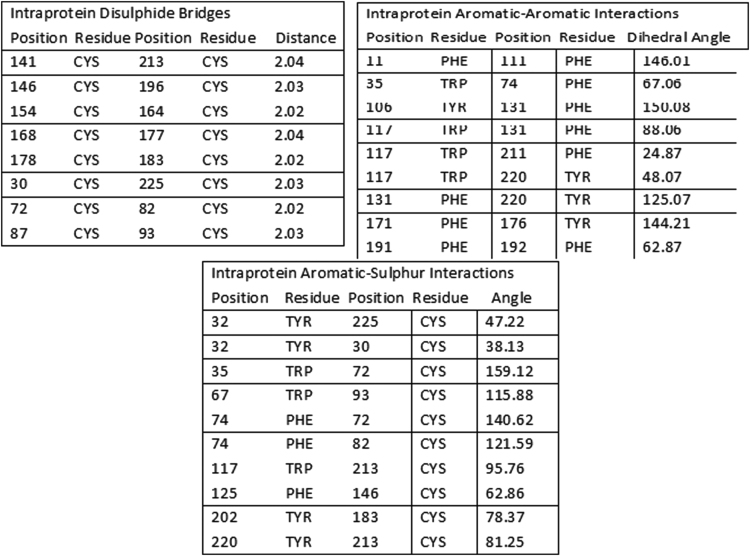
Different interactions of modeled NP24 protein.

To understand the model quality and recognition of errors in the model, PROSA tool was used. PROSA result shows overall model quality and local model quality. Quality has been assessed using Z-score. Obtained Z-score -4.43 lies within the range (Fig. 5). Furthermore, we have aligned the model structure with that of the crystal structure of NP24-I (PDB ID: 2i0w) using the DaliLite tool. Both structures have a greater identity with Z-score equal to 35.4 and RMS of C-alpha value equal to 0.6 (Fig. 6). Structural analysis of modeled protein shows that NP24-I has five helices, 19 strands, 34 turns, 128 hydrogen bonds and eight disulphide bridges which stabilized the entire structure. All interactions including disulphide bridges are shown in Fig. 7. It was observed that there were 10 clefts. Out of 10 clefts, four have higher volumes which are more significant. It was also observed that different residues present in protein NP24 according to their properties of an amino acid. The first cleft is the biggest cleft with the volume 2582 Å contains one cysteine molecule, three positive and two negatively charged atoms with more number of aliphatic residues. Whereas the second cleft with 1766 2582 Å volume contains two cysteine molecules which show a more stabilized structure. An important feature of the 2nd cleft is the presence of a higher number of neutral and aromatic residues. IgE epitopes present in protein NP24 predicted by AlgPred tool show stick-like shape.

## 8 IgE Epitope mapping using IEDB׳s homology mapping tool

Predicted IgE epitopes in NP24 protein were mapped on 3D structures and the sequence of some TLPs. It shows source sequences appeared in the regions that are similar to the epitope sequence. The epitope location annotated by IEDB were highlighted by green and orange color which indicate perfect sequence matches while other matches (identity > 80%, overlap >= 80% and no more than one gap) indicated in light grey color. Epitope mapping resulted in 19 hits which had epitope matches to the structures. These 19 hits have sequence similarity greater than 39%. This study also shows number of residues of the epitope are exactly present in the homologous structures of proteins.

Pathogenesis-related (PR) group of protein is one of the specific plant proteins group especially found in vegetables which has the number of types from ranged from PR1 to PR 17 [29], [30]. Protein sequences which show maximum similarity to Thaumatin protein (TP) belonged to PR-5 group. Most of the PR families show functional diversity in allergenicity and kinase function despite their sequence similarity [31].

Current understanding of the cross-reactivity of food allergens from Tomato fruit is very rare. NP24 protein alignment and analysis using 80-mer window methods (FARRP and Allermatch) shows that Kiwi, Bell Pepper, Banana, Wheat and Peach show > 60% identity with Thaumatin-like proteins (TLPs) from plant food (vegetables). The pollen TLPs considerable similarity numbers of pollen TLP were very few [32]. The number of 8-mer matches with NP24 protein by FARRP and Allermatch was found to be high for Bell Pepper, Olive and Kiwi.

AlgPred epitope analysis of NP24 protein indicates the presence of seven (7) different types of IgE epitopes in the homologous sequences. Among TLPs, some protein sequences very few have only four epitopes and others are devoid of epitopes. IgE epitopes 1 and 2 are the predominant of food TLPs. On contrary, IgE epitopes 1, 2 and 3 frequently in most pollen TLPs. Secondary structure analysis shows that structure of NP24 protein is much more similar to other TLPs. The secondary structure of NP24 shows high percentages of amino acids such as glycine 28/247 (11.33%), threonine 25/247 (10.12%) and proline 21/247 (8.5%).

Mapping of NP24 protein epitopes: The two predicted IgE epitopes (1 and 2) of NP24 protein have been mapped. It was observed that some residues in IgE epitopes one (1) and two (2) were buried. But, most of the residues were readily accessible and specific IgE of protein NP24 produce allergic reactions.

The protein NP24 is a commonly found component in Tomato fruits and Spinach leaves. It shows the close match with allergenic TLPs of Capsicum, Kiwi, White Cedar, *Cupressus sempervirens* and Banana suggesting cross allergic reaction. Two unique IgE epitope of NP24 protein were identified viz. Epitope 1 (AAGTASARFWGRT), Epitope 2 (TFDASGKGSCQTG) at positions 58–70 & 73–85 position respectively. Amongst seven IgE epitopes, the epitope number 1, 2 & 3 showed a greater degree of conservancy within the homologous sequence to NP24 protein. Phylogenetic analysis of protein NP24 with other TLPs revealed that Capsicum shows highest allergic cross-reactivity with Tomato fruit NP24 protein.
